# *Aerobiology*—A New Open Access Journal

**DOI:** 10.3390/aerobiology1010001

**Published:** 2023-08-03

**Authors:** Chad J. Roy

**Affiliations:** Department of Microbiology and Immunology, School of Medicine, Tulane University, New Orleans, LA 70118, USA

It is simultaneously professionally humbling and an absolute pleasure to be associated with the launch of a new open access journal, with added emphasis in a scientific field as rich and diverse as aerobiology. The field of aerobiology encompasses the living microbiologically diverse world in our collective atmosphere, spanning to both the ecological phenomena and the anthropomorphic effects of a shared biology in the air. The conception of the Journal is intended to be unique amongst peer publications—the scope of *Aerobiology* covers both the ecological study of bioaerosols and the subsequent interaction in the natural and built environment but also the ultimate disposition in biological systems [1], with emphasis on purported human health effects from exposure and transmission studies. Collectively, the subject matter encompassed under this broad scope will provide a focal source on aerobiology for many years to come.

There is a strong argument to be made for an aerobiology Journal whose scope spans both ecological and human health. The microbiological diversity in what is transported in our collective atmosphere drives many processes in the ecology of the planet and how our ever-changing environment impacts human health. Identification and characterization of the changes associated with the airborne transport of bioaerosols provide touchstone data on the effects of climate change on the planetary environment [2], agricultural crop science [3], trends in global microbial biodiversity [4], and the seasonal regionality of allergens [5]. Similarly, centric information on the aerosol efficiencies of potentially infectious microorganisms [6], the dispersity of naturally synthesized biological toxins [7], and transmission dynamics of endemic and emerging pathogens [8] are centrally thematic to many of the parameters measured and communicated; the curation of aerobiology datasets generated from both the life sciences and the health sciences share a unique kinship yet touch many and diverse areas of science.

The COVID-19 pandemic provides an idealized exemplar of how rapid dissemination of scientific information in specific areas of inquiry was needed in critical decision-making and ultimately adding and expanding academic knowledge of the subject matter. In the case of the world emergence of SARS-CoV-2, the recognition that the virus was indeed airborne was built upon data methods and critical thinking that decidedly fell within the field of aerobiology. It was only after the understanding that the emergent virus remains replication competent in aerosol suspensions that longer distance transmission was possible [9,10], galvanizing the concept of aerosol-mediated infection in susceptible populations. Now in retrospect, much of the experimental configurations, studies, and data curation associated with addressing the transmission question in the SARS-CoV-2 example relied heavily upon the basic aerobiology practices used in ecological studies. This is a clear demonstration of the commonalities in modern aerobiological studies and provides a clear rationale for combining the life and health sciences under a single journal aegis of *Aerobiology*.

The Journal *Aerobiology* (ISSN 2813-5075) [11] holds great potential as an informational source for those interested in reading and contributing to the knowledge base of the continuous and dynamic interactions between the microbial world and our collective atmosphere. It is our hope that by the establishment of *Aerobiology*, the journal will provide an essential venue for the healthy exchange of prevailing theory and associated discourse in this much understudied subject matter area. *Aerobiology* fills a void in current publications as the Journals scope spans both the life and health sciences, whereas other aerobiology-centric journals mainly cover life sciences. Those of us engaged in the curation of aerobiology data should care about the growth and vitality of this publication and ensure its success by contributing our work without delay. An exceptional group comprises the growing editorial board of *Aerobiology*, whose deep expertise spans numerous applied subjects in this exciting field of study. There is palpable excitement at the prospect of submission and review of submissions from a diverse group of engaged contributors throughout the scientific world community. I would like to thank everyone for their continued interest in the field of aerobiology, and I am looking forward to working towards making *Aerobiology* one of the marquee open access publications in the sciences.
